# Predictors for Antipsychotic Dosage Change in the First Year of Treatment in Schizophrenia Spectrum and Bipolar Disorders

**DOI:** 10.3389/fpsyt.2019.00649

**Published:** 2019-09-10

**Authors:** Petter Andreas Ringen, Elina Johanna Reponen, Trude Seselie Jahr Vedal, Ola A. Andreassen, Nils Eiel Steen, Ingrid Melle

**Affiliations:** ^1^Division of Mental Health and Addiction, Oslo University Hospital, Oslo, Norway; ^2^Norwegian Centre for Mental Disorders Research, Faculty of Medicine, University of Oslo, Oslo, Norway

**Keywords:** antipsychotics, schizophrenia, bipolar disorder, prescription, dosage

## Abstract

**Background:** Use of antipsychotic medication is central in the treatment of psychotic disorders. However, there is limited knowledge about prescription practice of antipsychotics in the critical early phase of these disorders. Clinical guidelines recommend low dosages, but no discontinuation of antipsychotic medication during the first year of treatment in first episode patients. The main aim of this study was to identify clinical predictors for dosage change or discontinuation of antipsychotics during this period.

**Methods:** A total of 426 antipsychotic-using patients with schizophrenia spectrum or bipolar disorder, including both a first treatment sample and a sample of patients with previous treated episodes (“multi-episode” sample) from the same diagnostic groups, underwent thorough clinical and sociodemographic assessment at study baseline and after 1 year. Prescribed dosage levels at baseline and follow-up and change in dosage or discontinuation of antipsychotics from baseline to follow-up were compared between groups, controlling for possible confounders.

**Results:** We found reduced dosages over the first year in both first treatment groups across diagnoses, but not in multi-episode groups. Weight increase predicted dosage reduction in the schizophrenia group, while the level of psychotic symptoms at baseline predicted dosage reduction in the bipolar group. We found higher baseline levels of antipsychotic use in the schizophrenia group than in the bipolar group.

**Conclusion:** We found indications of a trans-diagnostic reduction of prescribed dosages of antipsychotics over the first year in treatment, but with different predictors for this reduction in the two diagnostic groups. The findings increase the understanding of drivers of early medication change in psychotic disorder.

## Background

Psychopharmacological agents are one of the main treatment approaches in clinical psychiatry. Antipsychotics (“neuroleptics”) were introduced for the treatment of schizophrenia in 1956, and have persisted as the cornerstone of treatment for schizophrenia and related psychotic disorders (1, 2). They also effectively reduce the psychotic symptoms of affective psychoses in manic or depressive episodes (3), and some are approved as mood stabilizers in bipolar disorder (4, 5). There is however growing controversy around the use of antipsychotics (6, 7), including criticism of inadequate side effect management (8–11).

Prospective studies show beneficial effects of earlier adequate treatment in psychotic disorders, and treatments given in the early phases of illness seem to be of particular importance for short- and long-term outcome (12–14). Studies also indicate that first-treatment patients are more sensitive to lower dosages of antipsychotics than multi-episode patients, both in their responses to and in their experiences of adverse effects (15). Use of unnecessarily high dosages may lead to adverse effects and negative opinions about antipsychotics, with long-term consequences for treatment compliance. New treatment guidelines recommend the use of monotherapy and low dosages for the first psychotic episode and subsequent maintenance therapy for schizophrenia spectrum disorders (2, 11, 16). Discontinuing antipsychotic treatment in the first year of treatment is not recommended. In clinical practice, the choice of which antipsychotic to use and the dosage to administer is highly individualized (17–19). Factors which may affect the choice of individual treatment include diagnosis, current symptomatology, insight into the illness, known side effects of the drug in question, degree of functional loss, as well as perceived adherence to medical advice (20, 21). There are no specific recommendations for antipsychotic treatment of patients with bipolar disorder.

The efficacy of current antipsychotics is dependent on their effects on the dopamine neurotransmitter system in the central nervous system. Since drug uptake, first-pass metabolism and passage over the blood–brain barrier are highly individual there is no fixed dose–response (22, 23), and antipsychotic treatments typically have an aspect of trial and error (24). This carries risks, both for the use of ineffective dosages over too long periods of time, and for the use of too high dosages causing unnecessary side effects. An increase in knowledge to guide the choice of first treatment in psychotic disorder is thus warranted. Identifying predictors of discontinuation or change in dosages of antipsychotics during the first year of treatment can help to better understand the mechanisms behind the individual decision-making processes. Such insight may be important in guiding the complex task of finding the optimal treatment.

The current study is based on a 1-year follow-up of comprehensively characterized patients with schizophrenia and bipolar disorder, including both first-treatment and multi-episode patients from the same catchment areas, and thus using the same treatment services. These services give treatment based on guidelines that recommend low dosages of antipsychotics for first-treatment schizophrenia for 2 years, but with no particular advice for the dosages and length of maintenance treatment in first-treatment bipolar disorder. The study had the following aims:

1. Are there differences in the use of antipsychotics, between first-treatment and multi- episode patients in patients with schizophrenia spectrum and bipolar disorder at study baseline?
2. What dosage changes or discontinuation rates of antipsychotic medication are there over the subsequent year? Do these predictors differ between patients with schizophrenia spectrum and bipolar disorder, or between first-treatment and multi- episode patients?
3. What are the predictors of changes in the dosages or discontinuation of antipsychotics, and are there different predictors between patients with schizophrenia spectrum and bipolar disorder, and between first-treatment and multi-episode patients?

## Materials and Methods

The current study is a component of the TOP (Thematically Organized Psychosis research) Study, which is approved by the Regional Committee for Medical Research Ethics and the Norwegian Data Inspectorate. Recruitment was done from 2003 until 2017 from major hospitals in the Oslo area, Norway. The TOP study comprises several smaller sub-studies. All first treatment patients are included in prospective cohorts with planned follow-up studies. Multi-episode patients were part of smaller follow-up studies, based on the focus of ongoing projects. The reasons for multi-episode patients to participate, or not to participate, in follow-up studies were thus administrative (based in project design and funding) with no identified selection bias involved. There were no significant differences in baseline demographic and clinical characteristics between multi-episode patients participating or not participating in follow-ups. For a more detailed description, see Faerden et al. (25), Hellvin et al. (26), and Kvitland et al. (27).

Inclusion criteria at baseline: age 17 to 67 years and meeting the DSM-IV criteria for a diagnosis of schizophrenia, schizophreniform disorder, schizoaffective disorder, psychotic disorder NOS, delusional disorder or bipolar I, II or NOS disorder. Exclusion criteria were: presence of a diagnosis of a developmental disorder, IQ < 70 or acquired brain damage (head injury with hospitalization), and lack of fluency in a Scandinavian language. There were no exclusion criteria based on course of illness, history of treatment or substance use. Patients were recruited consecutively from in- and outpatient psychiatric units in the collaborating hospitals. There were no other treatment organizations serving these areas, allowing for a high degree of representation for participating patients. For details, see Ringen et al. (28). Each patient was referred to the project by their treating clinician, after an initial evaluation of their eligibility and ability to give informed consent. Emphasis was put on recruiting all patients regardless of the level of adherence to their respective treatment programs. All patients gave written informed consent to participation and for follow-up. The assessments were conducted by trained clinicians working as research fellows (MDs or clinical psychologists). The recruitment teams were primarily based in the outpatient clinics, where patients are transferred for treatment after the acute illness phases. The study thus mainly includes patients who were symptomatically stable at the point of baseline assessments.

### Assessments of Diagnosis, Onset of Illness, Treatment History and Sociodemographics at First Assessment; Creating of Groups

Diagnosis, onset of illness and treatment history at baseline were established using the Structural Clinical Instrument of Diagnosis for DSM-IV axis I disorders (SCID – I), modules A–E, with the aid of medical charts (29). All interviewers completed a training course in SCID assessment based on the training program at the University of California Los Angeles (30) and participated in regular diagnostic consensus meetings led by a clinically well-experienced professor of psychiatry. To evaluate reliability of actual study interviews, a stratified random sample was drawn, consisting of cases from every assessment staff member. Anonymous vignettes describing symptoms and development of the illness were then rated by two experts blind to the study ratings. For the 28 vignettes evaluated, the overall agreement for the nine DSM-IV diagnostic categories was 82% and the overall Kappa 0.77 (95% CI: 0.60–0.94).

The duration of untreated illness (DUI) was defined as the time (in weeks) from the onset of the first SCID verified illness episode to the start of use of adequate medication. For Bipolar disorder specifically, DUI was defined as time from first affective episode (regardless of polarity), to the start of adequate treatment, defined as either antipsychotic or mood-stabilizing medication for mania or mixed episodes (in appropriate dosages for minimum 6 weeks) according to available treatment guidelines for BD I (31).

Data was collected on marital status, occupational status, and educational level. Four groups were defined: “First treatment patients” were defined based on treatment history as patients receiving their first adequate treatment of the disorder in question within the last 12 months. “Schizophrenia spectrum” (SS) included schizophrenia, schizophreniform disorder, schizoaffective disorder, psychotic disorder NOS and delusional disorder and was further divided into “First Treatment Schizophrenia Spectrum” and “Multi-episode Schizophrenia Spectrum” based on treatment history. “Bipolar disorder” (BD) included bipolar disorder I, II and NOS and was divided into “First Treatment Bipolar Disorder” and “Multi-episode Bipolar Disorder.”

### Assessment of Medication, Functioning, Symptoms and Socio-Demographical Characteristics at First Assessment (Baseline) and 12 Months Follow-Up

All patients were assessed at first recruitment (“Baseline”) and 12 months later. At both time points, information on type and dosage of all antipsychotic medication was collected. Defined daily doses (DDD) were defined according to the WHO criteria (32, 33). For comparison of DDDs and Chlorpromazine equivalents, see **Table 1**. The ratios of currently prescribed daily dosage of an antipsychotic (PDD) and the corresponding DDD (PDD/DDD) were calculated for each prescribed antipsychotic. The sum of all PDD/DDD ratios for each participant was used as an estimate of current load of antipsychotics across different types of drugs. “PDD/DDD change” was created by subtracting PDD/DDD at 12 months follow-up from PDD/DDD at baseline (including cases not using antipsychotics, i.e., PDD/DDD = 0, at follow-up). The *Udvalg for Kliniske Undersøgelser* (UKU) side effect rating scale (34) was used to measure type and severity of side effects. All items in the UKU scale were scored from 0 to 3, where 0 indicated no side-effect, and scores 1–3 indicated presence of side-effect with increasing severity.

**Table 1:** Prescribed antipsychotics and comparison of defined daily dose and chlorpromazine equivalents.

	Defined daily dose Dose (mg/day)	CPZ eqv* Dose (mg/day)
Chlorpromazine	300.0	100.0
Haloperidol	8.0	1.6
Perphenazine	30.0	6.8
Zuclopenthixol	30.0	*na*
Amisulpride	400.0	*na*
Aripiprazole	15.0	8.0
Paliperidone	6.0	*na*
Olanzapine	10.0	5.3
Quetiapine	400.0	175.5
Risperidone	5.0	1.2
Ziprasidone	80.0	62.6
Clozapine	300.0	138.8

*Chlorpromazine equivalents, linear equations (35). na, not available.

Insight was measured by the Birchwood Insight scale (36) items 2 and 8, and low levels of insight were defined as a score of 3 or higher. Level of physical activity was assessed by the clinicians as “light,” “medium,” or “heavy.” Patients were interviewed about substance use prior to first assessment and in the follow-up period based on a common semi-structured interview form and from section “E” of the SCID (29). Current global functioning and symptoms were assessed by the Global Assessment of Functioning Scale (GAF), using the split version of GAF, with separate scores for symptoms and functioning (37). Current psychotic symptoms were assessed by the Positive and Negative Syndrome Scale (PANSS) (38). Current depressive symptoms were measured with the Inventory of Depressive Symptoms – Clinician rated (IDS-C) (39), and current manic symptoms were rated with the Young Mania Rating Scale (YMRS) (40). The Alcohol Use Disorders Identification Test (AUDIT) (41) was used to identify problematic alcohol use.

The inter-rater reliability of the symptom assessments in the TOP study have been shown to be good with an Intraclass Coefficient (ICC) (42) of 0.82 for PANSS positive symptoms and 0.86 for GAF (28).

### Statistics

All analyses were performed using the Statistical Package for the Social Sciences (SPSS version 25.0, SPSS Inc., Chicago, IL, USA). Differences between categorical variables were analyzed using chi square tests. Differences between normally distributed continuous variables were analyzed using univariate analyses of variance with *post hoc* Bonferroni corrections and paired t-tests as appropriate. Significance level was set to 0.05, two-tailed.

A two-way between-groups analysis of variance was conducted to explore the impact of diagnostic group (schizophrenia spectrum or bipolar disorder spectrum) and treatment group (first treatment or multi-episode) on change in dosage of antipsychotics. To identify predictors for change in dosages of antipsychotics from first treatment we performed a series of follow-up multivariate analyses for each group. We here used multiple linear regression analyses for normally distributed dependent variables, with independents entered hierarchically in several blocks. Age and sex were selected as priori independent variables, in addition to baseline measures regarded as plausible predictive factors for inducing change, including measures of common side-effects, insight and reported compliance. Additional putative predictors were added based on findings of significant bivariate associations to changes in dosage of antipsychotic medication in the current sample. The assumption of a linear relationship was evaluated based on examinations of residual plots for each analysis, and on examination of influential observations based on leverages and Cox distances. The final model with the best fits is presented in the paper.

## Results

A total of 426 patients were included in the current study, with assessments both at baseline and follow-up. Their demographic and clinical characteristics at baseline are described in **Table 2**. Out of these, 136 patients did not use antipsychotics at baseline (First Treatment Schizophrenia Spectrum: 40 (21% within the diagnostic group); First Treatment Bipolar Disorder: 47 (47% within the diagnostic group); Multi-episode Schizophrenia Spectrum: 10 (15% within the diagnostic group); Multi-episode Bipolar Disorder: 39 (57% within the diagnostic group)). Eight patients did not have reliable information for antipsychotic use at follow-up. For the 286 patients with information on dosage of antipsychotics at baseline, the schizophrenia spectrum patients used significantly higher dosages of antipsychotics than bipolar disorder patients (**Table 3**). There were no significant differences between first-episode and multi-episode groups.

**Table 2:** Demographic and clinical characteristics of groups at first assessment.

	Multi-episode schizophrenia spectrum (N = 69)			Multi-episode bipolar disorder (N = 69)			First treatment schizophrenia spectrum (N = 187)			First treatment Bipolar disorder (N = 101)		
	n	Mean	SD	n	Mean	SD	n	Mean	SD	n	Mean	SD
Age, years	69	33.2	1.2	69	35.4	11.8	187	26.9	7.5	101	3.3	9.7
Premorbid functioning	66	0.3	0.2	68	0.2	0.2	181	0.23	0.18	101	0.18	0.16
Education, years	69	13.0	2.6	68	14.4	3.3	187	13.1	2.9	101	14.6	2.7
DUI, weeks	9	121.2	132.3	6	21.5	4.0	185	119.1	199.4	54	43.1	134.3
Age at onset*, years	67	27.0	9.4	31	28.5	11.3	182	23.8	7.3	66	27.0	8.8
Age at first medication*, years	61	28.2	9.5	31	3.7	12.2	166	25.7	7.0	56	29.3	9.6
Number of suicide attempts	69	1.0	2.8	69	0.6	1.4	183	0.5	1.5	100	0.5	1.6
BMI, kg/m^2^	67	26.4	5.0	69	25.8	4.3	181	24.6	4.3	98	24.9	4.2
Audit	20	3.5	5.9	16	0.7	1.9	177	5.8	9.5	87	3.4	6.4
Audit	17	7.2	7.0	10	5.9	4.6	172	7.2	7.2	83	8.9	6.8
GAF-Symptoms	69	45.0	12.0	69	57.6	9.9	187	43.0	12.3	101	57.9	11.5
GAF-Functioning	69	47.2	11.5	69	55.9	11.0	187	45.2	12.8	101	54.2	11.9
PANSS positive symptoms	69	14.4	5.6	69	9.5	2.8	187	15.1	4.9	101	9.9	3.6
PANSS negative symptoms	69	15.6	6.3	69	1.7	3.7	187	14.9	6.0	101	1.0	3.1
PANSS general symptoms	69	31.8	9.9	69	26.5	5.5	186	32.1	7.0	101	25.9	5.3
IDS depressive symptoms	65	18.3	13.0	66	15.2	1.5	106	17.1	12.7	95	16.8	11.5
YMRS manic symptoms	67	4.9	4.6	69	2.9	3.6	165	5.7	4.9	101	3.8	5.3
Side effects of medication	55	11.3	7.8	61	9.0	5.8	148	12.8	9.5	78	15.3	11.3
	n	%		n	%		n	%		n	%	
Male	41	59.4		31	44.9		115	61.5		41	40.6	
European (Caucasian)	57	82.6		60	87.0		132	70.6		85	84.2	
Never married and single	49	71.0		39	56.5		141	75.4.		57	56.4	
Daily tobacco use	45	65.2		36	52.2		89	47.6		51	50.5	
5+ cups of coffee daily	35	50.7		19	27.5		32	17.1		28	27.8	
Cannabis use past 14 days	7	10.1		2	2.9		23	12.4		11	10.9	
Medium/high physical activity	16	40.0		17	39.5		49	33.3		36	43.3	
BIS: No need for medication	40	60.6		38	56.7		63	40.1		50	51.5	
BIS: Low insight in illness	40	60.6		45	67.2		64	40.8		64	66.0	

*Of symptoms. SD, standard deviation; DUI, duration of untreated illness; BMI, body mass index; GAF, Global Assessment of Functioning; PANSS, Positive and Negative Syndrome Scale; IDS, Inventory of Depressive Symptoms; YMRS, Young Mania Rating Scale; BIS, Birchwood Insight Scale.

**Table 3:** Total dosage of prescribed antipsychotics per defined daily dose (PDD/DDD) of all antipsychotics in use at baseline. N = 286 with information on PDD/DDD at baseline.

	n	Mean	SD	Post hoc*
First treatment schizophrenia spectrum	147	1.32	0.85	vs FTBD: 0.011, vs MEBD: 0.001
First treatment bipolar disorder	53	0.92	0.65	
Multi episode schizophrenia spectrum	56	1.42	0.89	vs FTBD: 0.007, vs MEBD: 0.001
Multi episode bipolar disorder	30	0.69	0.45	

*ANOVA with Bonferroni correction. S.D., Standard Deviation; FTBD, First Treatment Bipolar Disorder; MEBD, Multi Episode Bipolar Disorder.

Use of antipsychotics and discontinuation rates for the different groups at baseline and follow-up are shown in **Table 4**. Of the 290 patients using antipsychotics at baseline, we had information on use of antipsychotics at follow-up for 282, of these 50 (18%) discontinued use. The difference in discontinuation rates between First Treatment Schizophrenia Spectrum and First Treatment Bipolar Disorder was statistically significant (x^2 =^ 4.6, p = 0.032). Thirteen (68%) of those discontinuing antipsychotics in the First Treatment Bipolar Disorder group had however changed to mood stabilizers at follow-up.

**Table 4:** Use of antipsychotic at baseline and follow-up.

	Full sample, N = 426		Use of AP at baseline and information on use of AP at follow-up for the same patient, n = 282
	Use of AP at baseline, n (%)	Use of AP at follow-up, n (%)	Discontinuation of AP, n (%)
First treatment schizophrenia spectrum	147 (78.6)	133 (74.3)	24 (16.8)
First treatment bipolar disorder	54 (53.5)	46 (49.5)	19 (36.5)
Multi-episode schizophrenia spectrum	59 (85.5)	58 (86.6)	2 (3.5)
Multi-episode bipolar disorder	30 (43.5)	28 (40.6)	5 (16.7)

*AP, antipsychotic.*

**Figure 1:**
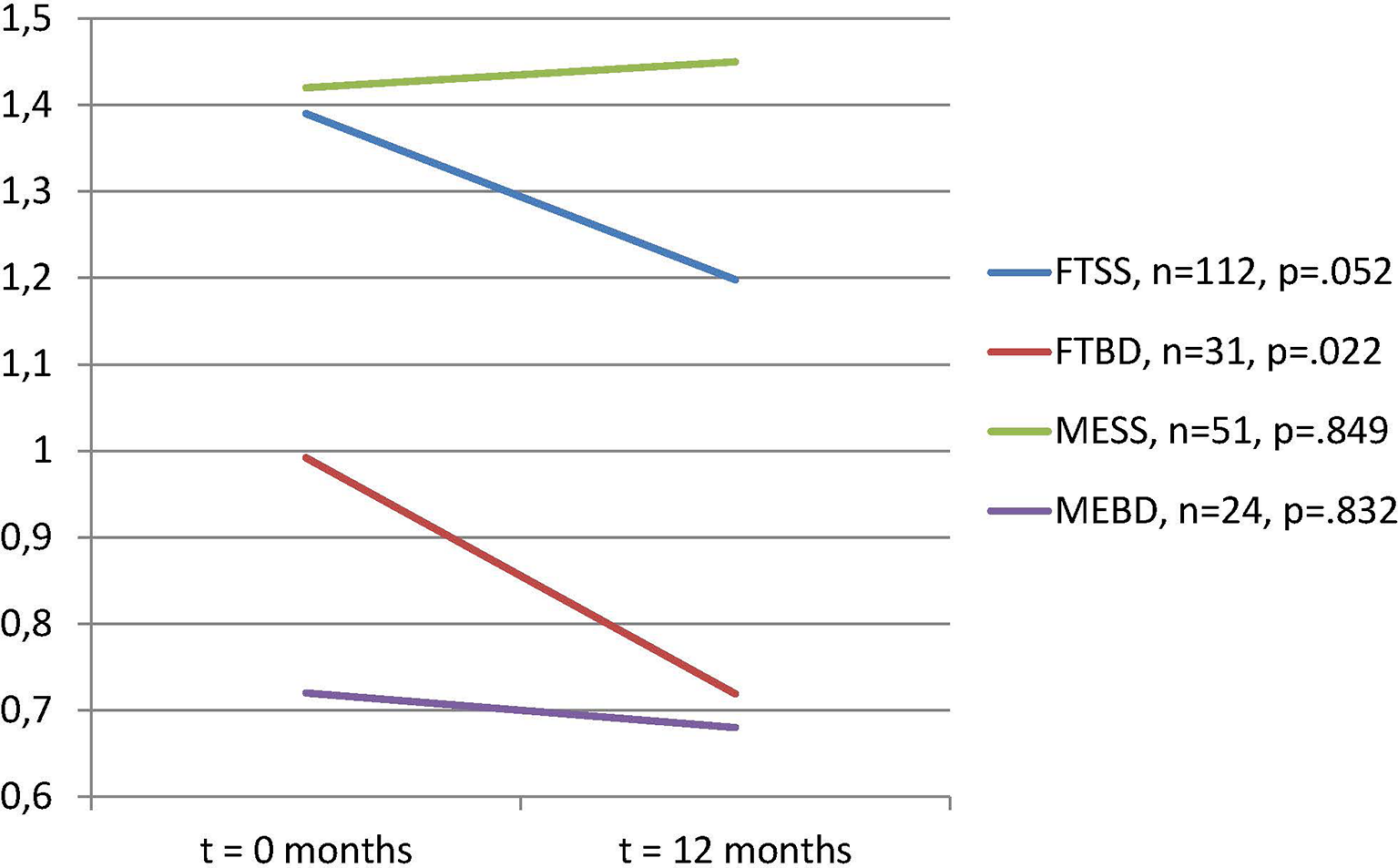
Total dosage of prescribed antipsychotics per defined daily dose (PDD/DDD) of all antipsychotics in use, paired samples of subjects with dosages >0 at t = 0. Paired t-tests. N = 218 with information on PDD/DDD and use at both time points.

There was a statistically significant reduction in antipsychotic dosage for both the First Treatment Schizophrenia Spectrum group and the First Treatment Bipolar Disorder group. There were no significant changes in antipsychotic use in the two multi-episode illness groups (**Figure 1**). A two-way between-groups analysis of variance with change in PDD/DDD ratios as dependent variable showed a statistically significant main effect for treatment group (first-treatment versus multi-episode) (F = 4.66, p = 0.032), however with a small effect size (ခη^2^0.02). There was no significant main effect for diagnostic group and no significant interaction effects.

Bivariate analyses showed the following group-wise significant associations with change in dosage of antipsychotics measured as PDD/DDD ratio: First Treatment Schizophrenia Spectrum: Age (+), PDD/DDD for all antipsychotics at baseline (−), UKU weight gain (−), UKU investigators’ assessment of global side effect load (−) and level of physical activity (−); First Treatment Bipolar Disorder: PDD/DDD for all antipsychotics at baseline (−), UKU hypokinesia (−), GAF-F (+), and PANSS-P scores (−). Since there were no changes for multi-episode patients, we did not do follow-up analyses for these groups.

Final combined models for the multivariate linear regressions analyses are presented in **Supplementary Tables 1A, B**. In First Treatment Schizophrenia Spectrum, a reduction in antipsychotic PDD/DDD ratio was significantly associated, with a small effect size, with baseline weight increase as a side effect of medication, as judged by the person conducting the assessment. In First Treatment Bipolar Disorder, a reduction in antipsychotic PDD/DDD ratio was significantly associated, with a small to medium effect size, with baseline higher levels of positive psychotic symptoms as measured by the PANSS.

The logistic regression analysis with discontinuation as the dependent variable showed significant contributions to the risk of discontinuation from higher GAF-F and alcohol use (AUDIT scores) at baseline in First Treatment Schizophrenia Spectrum, and from increased age in First Treatment Bipolar Disorder, significant odds ratios were in the range from 1.06 to 1.11 (**Supplementary Tables 2A, B**).

## Discussion

As expected, and in line with current clinical recommendations (43, 44), we found that patients with schizophrenia spectrum disorders used higher dosages of antipsychotics compared to patients with bipolar disorders in both first-treatment and multi-episode groups. Contrary to clinical recommendations, we did not find that first-treatment patients used lower dosages at baseline as compared to multi-episode patients in both diagnostic groups. However, both first treatment groups showed significant reductions in dosages of antipsychotics over the first year of treatment, while both multi-episode groups did not show significant change.

In First Treatment Schizophrenia Spectrum, weight gain at baseline was a statistically significant predictor of dosage reduction over the first year of treatment. Although the effect size was modest, the association between weight gain and dosage reduction may be taken as an indication of awareness of the risks associated with obesity in this patient group, an aspect which is receiving increasing focus in clinical guidelines (2, 45).

In the First Treatment Bipolar Disorder group, the baseline level of positive psychotic symptoms predicted dosage reduction of antipsychotics, although with a small to moderate effect size. The higher dosages of antipsychotics in the First Treatment Bipolar Disorder compared to the Multi-episode Bipolar Disorder group at baseline could thus partly be explained by the first treatment patients being closer in time to an acute phase with high symptom levels. Antipsychotics are recommended for the acute phase of mania (44), and the high dosages observed could be a transient response to treatment needs in this phase and thus in line with main guidelines (1, 2, 5). Taken together, our findings point to diagnostic-specific associations with dosage reductions in first treatment patients.

The discontinuation rates found in the First Treatment Schizophrenia Spectrum group are in line with previous findings (46). We found that high levels of functioning and problematic use of alcohol at baseline significantly predicted discontinuation in this group. These findings are clinically meaningful, as alcohol abuse has been shown to affect adherence to medical advice (45). To the best of our knowledge, there are no previous reports of discontinuations rates of antipsychotics in First Treatment Bipolar Disorder. The rate in this group is higher than that observed in the other three groups, again indicating that the medication at baseline is a transient response to acute mania. In the First Treatment Bipolar Disorder group we found that increased age was a significant predictor of discontinuation of antipsychotics. Increased age is usually associated with improved adherence to treatment; however, in this case the patients discontinued antipsychotic treatment and changed to other psychopharmacological agents. A possible explanation could be that clinicians felt more confident of their diagnosis of BD in the older patients and thus were more prone to change to mood stabilizing medication for secondary prevention.

In this context, we should note that adherence to prescribed medication is a major challenge for first episode patients (47, 48). In addition, previous studies have found that physicians’ adherence to guidelines is adequate in the initial phase of treatment but reduces over time (49). The recommendation of low dosages for first-treatment phases also pertains to the acute phases of illness, and previous studies indicate that lower acute phase dosages is an achievable goal (50). Our findings may thus indicate that early dosage practices are more driven by acute phase symptoms than by guideline recommendations. Relapse prevention dosages are however in line with recommendations, with a particular emphasis on risk associated with obesity in First Treatment Schizophrenia Spectrum. In First Treatment Bipolar Disorder, antipsychotics appear to be used mainly as an acute phase treatment and not as relapse prevention.

The main strength of the current study is the well characterized and relatively large prospective sample of first-treatment patients with, both schizophrenia- and bipolar spectrum disorders followed over the early treatment phase. The catchment area based and consecutive sampling procedure, including both in-and outpatient treatment services, gives the sample a high degree of representation. The study also has some limitations. Although our cohorts of first treatment patients are large compared to other studies of bipolar disorders, some of the subgroups were relatively small which may increase the risk of type II errors. The study also used cross-sectional assessments at two time points, and there were thus restricted possibilities for a temporal sequencing of events in the follow-up period.

In conclusion, a statistically significant reduction in dosages over the first 12 months of treatment was associated with early medication-related weight increase in first treatment schizophrenia, and with higher levels of psychotic symptoms at baseline in first treatment bipolar disorder. Our findings thus document potentially clinically meaningful diagnostic differences in patterns of prescription and adaptive prescription changes in the early treatment phases of psychotic disorders, and add to the understanding of what drives early antipsychotic dosage change. We did not find indications of lower dosages of antipsychotics at baseline in first-treatment patients compared to multi-episode patients across diagnostic groups. Further, our findings emphasize the risks for discontinuation associated with all types of substance abuse, including alcohol. There is a need for more specific treatment recommendations for the use of antipsychotics in the early treated phases of bipolar disorder. Further studies should preferably investigate motivations for medication change for the physicians, in addition to patients.

## Ethics Statement

All subjects gave written informed consent in accordance with the Declaration of Helsinki. The protocol was approved by The Regional Committee on Research Ethics of South Eastern Norway.

## Author Contributions

PR and IM contributed to the conception and design of the study, performed the statistical analysis, and wrote the first draft of the manuscript. PR organized the database. ER, TV, OA, and NS wrote sections of the manuscript. All authors contributed to manuscript revision, and read and approved the submitted version.

## Funding

The study received funding from the Oslo University Hospital, the Regional Health Authority South Eastern Norway (Grant #2014-102) and the Research Council of Norway (Grant #223273).

## Conflict of Interest Statement

OA received speakers’ honoraria from Lundbeck.

The remaining authors declare that the research was conducted in the absence of any commercial or financial relationships that could be construed as a potential conflict of interest.
