# Supplementary material for: Predictors for Antipsychotic Dosage Change in the First Year of Treatment in Schizophrenia Spectrum and Bipolar Disorders
Source: Front Psychiatry. 2019 Sep 10;10:649. doi: 10.3389/fpsyt.2019.00649 (PMC6747902; doi:10.3389/fpsyt.2019.00649)
Supplement: Supplementary file 1 [file DataSheet_1.docx]

SUPPLEMENTARY MATERIAL

**Supplementary Table 1a. Linear regression: Prediction of change in PDD/DD ratio for first treatment schizophrenia spectrum disorder**

CI: Confidence Interval; UKU: *Udvalg for Kliniske Undersøgelser* side effect rating scale; PANSS: Positive and Negative Syndrome Scale

|  | Block model summary for each step | |  | |  |  |  |
| --- | --- | --- | --- | --- | --- | --- | --- |
| N=104 |  |  |  |  | |  |  |
| Block No. Variable | R2 Change | F Change | T | Beta | | 95% CI of B | P Value |
| Constant | … | … | -.550 | -.276 | | -1.270 to .718 | .583 |
| 1. Age | .036 | 3.803 | 1.472 | .018 | | -.006 to .043 | .144 |
| 2. UKU, Weight increase | .107 | 8.062 | -3.037 | -.280 | | -.463 to -.097 | .003 |
| 3. UKU, Hypokinesia | .003 | .321 | .673 | .128 | | -.250 to .506 | .503 |
| 4. PANSS, positive symptoms | .001 | .017 | -.333 | -.007 | | -.047 to -033 | .740 |
| 5. Medium or heavy physical activity | .035 | 3.980 | -1.995 | -.420 | | -.839 to -.002 | .049 |
| *Adjusted R square of final model: .101* |  |  |  |  | |  |  |

**Supplementary Table 1b. Linear regression: Prediction of change in PDD/DD ratio for first treatment bipolar disorder**

CI: Confidence Interval; UKU: *Udvalg for Kliniske Undersøgelser* side effect rating scale; PANSS: Positive and Negative Syndrome Scale

|  | Block model summary for each step | |  | |  |  |  |
| --- | --- | --- | --- | --- | --- | --- | --- |
| N=50 |  |  |  |  | |  |  |
| Block No. Variable | R2 Change | F Change | T | Beta | | 95% CI of B | P Value |
| Constant | … | … | 1.799 | .927 | | -.111 to 1.965 | .079 |
| 1. Age | .001 | .008 | -1.097 | -.011 | | -.033 to .010 | .279 |
| 2. UKU, Weight increase | .001 | .007 | -.607 | -.074 | | -.321 to .172 | .547 |
| 3. UKU, Hypokinesia | .133 | 7.061 | -1.368 | -.354 | | -.875 to .167 | .178 |
| 4. PANSS, positive symptoms | .127 | 7.761 | -2.798 | -.076 | | -.131 to -021 | .008 |
| 5. Medium or heavy physical activity | .005 | .290 | -.593 | -.109 | | -.516 to .298 | .593 |
| *Adjusted R square of final model: .182* |  |  |  |  | |  |  |

UKU: *Udvalg for Kliniske Undersøgelser* side effect rating scale; PANSS: Positive and Negative Syndrome Scale

**Supplementary Table 2a. Logistic regression with discontinuation of antipsychotics as dependent variable, first treatment schizophrenia spectrum disorder**

S.E.: Standard Error. OR: Odds Ratio. CI: Confidence Interval. GAF: Global Assessment of Functioning (split version): Functioning. AUDIT: Alcohol Use Disorders Identification Test.

| N=133 | B | S.E. of B. | OR | 95% CI for OR | P value |
| --- | --- | --- | --- | --- | --- |
| *Constant* | -3.34 | 1.62 | .04 | n.a. | .039 |
| Sex | -.58 | .55 | .56 | .19 - 1.63 | .288 |
| Age | -.03 | .04 | .97 | .89 - 1.05 | .449 |
| GAF F | .06 | .02 | 1.06 | 1.02 - 1.10 | .001 |
| AUDIT | .07 | .03 | 1.07 | 1.01 - 1.14 | .028 |

**Supplementary Table 2b. Logistic regression with discontinuation of antipsychotics as dependent variable, first treatment bipolar disorder**

S.E.: Standard Error. OR: Odds Ratio. CI: Confidence Interval. GAF F: Global Assessment of Functioning (split version): Functioning. AUDIT: Alcohol Use Disorders Identification Test.

| N=43 | B | S.E. of B | OR | 95% CI for OR | P value |
| --- | --- | --- | --- | --- | --- |
| *Constant* | -3.68 | 2.58 | .03 | n.a. | .154 |
| Sex | .50 | .86 | 1.64 | .31-8.78 | .563 |
| Age | .11 | .04 | 1.11 | 1.03-1.20 | .007 |
| GAF F | -.03 | .04 | .98 | .90 - 1.05 | .518 |
| AUDIT | .04 | .05 | 1.04 | .93 - 1.15 | .514 |
